# Novel Viral Vector Systems for Gene Therapy

**DOI:** 10.3390/v2041002

**Published:** 2010-04-14

**Authors:** Daniel Stone

**Affiliations:** Quantitative Biosciences Institute, Department of Chemical Engineering, University of California, Berkeley, CA 94720, USA; E-Mail: stone.d@berkeley.edu; Tel.: +1-510-642-4923; Fax: +1-510-642-5198

## Introduction

Over the last three decades, interest in the field of gene therapy seems to have fluctuated between hot and cold. Encouraging pre-clinical and clinical data has demonstrated the potential of genetic therapies and yet setbacks in clinical trials have cast doubts in some minds over the clinical future of gene therapy [1–3]. In the last two years, a number of studies have demonstrated therapeutic benefits in clinical trials aimed towards specific monogenetic disorders [4–6], and this has brought renewed optimism to the field.

Loosely defined, gene therapy is the process of treating a particular disease through the introduction of genetic material in order to elicit a therapeutic benefit. While the genetic mutations underlying various diseases are well understood, delivering a corrective gene to the diseased organs/tissues remains a formidable challenge. Ultimately, the inability to successfully and specifically target enough of the correct cell types will limit the prospects of a beneficial outcome. Therefore, gene therapy strategies remain limited by the chosen method of gene delivery system.

While several methods of gene transfer have been used in genetic therapies, the process of virus-mediated gene transfer has been far and away the most popular within the gene therapy community [7]. As viruses can be manipulated with relative ease and through evolution have attained the ability to efficiently deliver their genomes to the nucleus of many different cell types and organs, they make good gene transfer vectors. For individual therapies the requirements of a viral vector differ according to the nature of the disease. While some therapies require long-term gene transfer, others require short-term or even regulated gene delivery. In some cases widespread gene transfer is a requirement, whereas localized gene transfer is preferred in others. Furthermore, gene packaging capacity and safety issues are also considerations when choosing which viral vector might be suitable for a given therapy. Although many different viruses have been modified to serve as gene transfer vectors, it is not always clear which is the best at meeting the requirements of a particular approach to treat a given disease.

This special issue introduces recent advances in the development of commonly used and lesser known viral vector systems. It demonstrates that there are multiple options open to researchers wanting to use viral vectors to treat specific diseases. Finally, it discusses some of the safety and toxicity concerns that face the use of viral vectors.

### Commonly used viral vectors

In gene therapy clinical trials the most commonly used gene delivery systems have been based on adenovirus (Ad), retrovirus, poxvirus, adeno-associated virus (AAV) and herpes simplex virus (HSV), which were cumulatively used in more than 66% of all clinical trials to date [7]. Initially, gene delivery systems were developed from these viruses since they are easily manipulated *in vitro* and have been studied in great detail. These viral vectors have a wide range of attributes that determine their suitability for different therapeutic applications.

Although Ad vectors have been widely used in a vast number of different pre-clinical applications, in the clinic they have mainly been considered for use in applications such as cancer gene therapies or vaccination that require short-term gene transfer to specific cell types and/or organs (For detailed Ad review see [8]). The lack of Ad integration machinery and host immune responses limits their use for long-term therapies, while the relative toxicity and high levels of human exposure make it undesirable for some therapies. In this issue *Wong et al.* discuss some of the developments made with oncolytic Ad vectors while *Seto et al.* discuss the use of bioinformatics-based approaches to identify and design novel Ad vectors.

Retrovirus vectors based on the murine leukemia oncoretrovirus (MLV) were the first to be used in gene marking and clinical trials in humans [9,10]. Retroviral vectors are able to efficiently infect dividing cells and stably integrate their genome to enable long term gene expression and this makes them good candidates for use in a number of therapies, including hematopoietic gene therapy. Unfortunately, tumorgenesis caused by integration of MLV sequences close to oncogenes in clinical trials has shown the limitations of such vectors [11], although newer generation retroviruses based on lentiviruses (including HIV) and foamy viruses have shown more favorable attributes, including the ability to infect non-dividing cells. In this issue *Castellani and Conese* introduce lentivirus vectors, and introduce their use in the development of therapies aimed at treating cystic fibrosis.

Poxviruses and in particular vaccinia virus have been widely used as gene therapy vectors, primarily as agents for vaccination (For detailed review see [12]). Through its use as a vaccine to eradicate smallpox, vaccinia has a well defined history of safe use as a vaccine vector in humans, and as such poxviruses have been developed for other genetic therapies that use a vaccination approach to treatment. More recently, poxviruses have also successfully been adapted for use as oncolytic vectors that are able to selectively kill tumor cells, and in this issue *Wong et al.* will discuss some of the developments made with oncolytic poxvirus vectors.

AAV is considered to be the safest viral vector system since it is based on a non-pathogenic human virus that can only replicate in the presence of a helper virus co-infection. For this reason, AAV is quickly becoming the viral vector of choice in clinical trials and particularly those which require long-term correction or enhancement of a genetic defect. While AAV has a good safety profile and is able to facilitate long-term gene expression to several organs, it is limited by a number of factors. The small genome of AAV limits the size of potential therapeutic genes that can be packaged, while there is a clear inability to efficiently infect certain cell types (such as hematopoietic stem cells) that are considered essential targets for a number of genetic therapies. For those of you with a desire to learn more about AAV there are a number of reviews that nicely introduce its advantages and disadvantages as a gene therapy vector [13,14]. Additionally, in this issue *Müther et al.* introduce a number of systems that utilize the integration machinery of AAV in hybrid viral vectors that target somatic integration.

As one of the largest human viruses, HSV is one of the most complex in terms of replication cycle and pathogenesis. Nevertheless, HSV has successfully been developed into a viral vector system that is primarily used in neurological therapies due to its natural neurotropism (For detailed review see [15]). While promising results have been seen using HSV vectors to treat neurodegenerative disorders or glioblastoma, the inherent toxicity of HSV has limited its use further. In this issue, *de Silva & Bowers* introduce a safer form of HSV amplicon vectors that express no viral genes.

### New types of viral vectors

In addition to the more commonly used viral vectors many other viral families have been used as gene transfer vectors. While most of them have not been used in clinical studies, there is an abundance of developmental and pre-clinical data demonstrating the various merits of these systems. Since the number of viral vector systems based on different viruses is vast, I have selected some interesting and novel systems to introduce in this special issue.

Several members of the alphavirus genus from the togoviridae family including the Semliki Forest virus (SFV), Sindbis virus (SIN) and Venezuelan Equine Encephalitis (VEE) have been developed into gene expression vectors in recent years. While their use has not been widespread and problems related to virus-induced toxicity have been seen, their prominent neurotropism has made them attractive for use in certain CNS-related gene therapies. *Kenneth Lundstrom* introduces the development of alphaviruses into effective gene transfer vector systems in this issue.

Instead of optimizing a given virus directly to improve its performance as a viral vector, the process of combining elements from different viruses or combining non-viral elements with viral elements to improve vector performance has gained a lot interest. These novel hybrid vector systems have shown a number of favorable attributes that make them attractive candidates for a number of gene therapies. In this issue, *Müther et al.* introduce a number of viral hybrid vector systems that have been developed for facilitating somatic integration of therapeutic genes.

As an alternative to switching viral families or genus in order to change the properties of viral vectors, several groups have proposed the encapsidation of viral particles with polymers that can serve a number of functions [16]. The primary function of the coat is to hide the virus from the host immune system upon *in vivo* administration, which in turn prevents degradation, increases vector circulation time and subsequently tissue transduction. Another function of polymer encapsidation could be to re-target a virus by first blanketing the particle to ablate its native tropism and then re-targeting the virus by conjugating targeting ligands directly to the polymer. In this issue *Wonganan & Croyle* illustrate the potential of such an approach, and introduce the use of poly(ethylene glycol) (PEG) as a virus encapsidation agent and describe the properties of PEGylated viruses in small and large animal models.

### Disease-based choice of viral vectors

The decision which viral vector to use for a given disease poses several problems to the gene threrapy researcher. Apart from the obvious need to transduce a particular cell type or organ, the route of administration, the total dose needed, and the potential toxicity of a viral vector must be taken into consideration. For some diseases, localized delivery to a specific organ is required whereas other diseases require body-wide gene delivery to multiple organs.

Irrespective of whether the aim is to treat a disease/disorder that manifests locally, such as Huntington’s disease, or a disease/disorder that manifests throughout the body, such as muscular dystrophy, researchers face a series of challenges. For different diseases/disorders the challenges vary and multiple different methods of gene transfer could be viable options for treatment. In this issue, from *Williams et al.*, and *Castellani & Conese* illustrate the various challenges one might have to face in deciding an approach to treat a particular disease/disorder. *Williams et al.* discuss the problems associated with trying to treat various forms of cardiovascular disease through gene therapy based approaches, while *Castellani & Conese* discuss the use of one particular vector type (lentiviral vectors) for the treatment of the monogeneic disorder cystic fibrosis.

### Safety concerns of viral vectors

With few exceptions, the wild-type forms of the many viruses used to generate viral vector systems are pathogenic agents that have their own inherent safety risks. Despite efforts to minimize the potential risks of individual viruses through genomic deletion, insertion or direct modification, it is not always possible to make a virus ‘safe’ without seriously affecting its ability to function as a useful viral vector. Therefore, it is extremely important to know the potential risks of a particular vector before using it, and to weigh the pros and cons between potential alternatives.

One of the main concerns following virus administration in a clinical setting is the rapid initiation of host responses to the vector immediately after delivery. Irrespective of whether a patient has had prior exposure to the virus being used as a vector, a number of native pathogen recognition systems exist in humans that can initiate a host immune/inflammatory response to the vector. This host response can be deleterious in a number of ways as it might hinder the effectiveness of the therapy [17] or, more importantly, the health of the patient [18]. To address the issue of host responses against the vector from a virological perspective, *Dmitry Shayakhmetov* discusses innate immune responses towards non-enveloped viruses, and how they impact the effectiveness of vectors used for gene delivery.

## Summary

Aside from their ability to cause disease in many species spanning, amongst others, reptiles, amphibians, fish, birds, and mammals, viruses offer the scientific researcher a wonderful series of tools for developing an understanding of disease and cell biology. Furthermore, they offer us methods of gene transfer that have the potential to be used beneficially in the treatment of many human diseases. It is my hope that this special issue introduces some of the more widely used viral vectors being used in gene therapy research, while also demonstrating that there are new vector systems available. Without the continuous development of improved and safer viral vectors, the advancement of genetic therapies will not continue.
